# Three cases of retained cuff related infection after manual pull removal of peritoneal dialysis catheter

**DOI:** 10.1080/0886022X.2020.1858872

**Published:** 2020-12-22

**Authors:** Suojian Zhang, Xu Zhang, Haitao Li, Zhiqiang Wei, Juan Cao

**Affiliations:** Department of Nephrology, Taixing People’s Hospital, Taizhou, Jiangsu, China

Dear Editor,

In China, the ‘pull technique’ is increasingly used for catheter removal in peritoneal dialysis (PD) patients. When this technique is employed, the cuff is retained after PD catheter removal. However, there are few reports of retained cuff-related infection after PD catheter removal. Here, we report the cases of three patients with retained cuff-related infection after catheter removal, who were cured by surgical resection and anti-infection therapy. All three patients received hemodialysis treatment after PD catheter removal.

## Case 1

**Figure 1.**
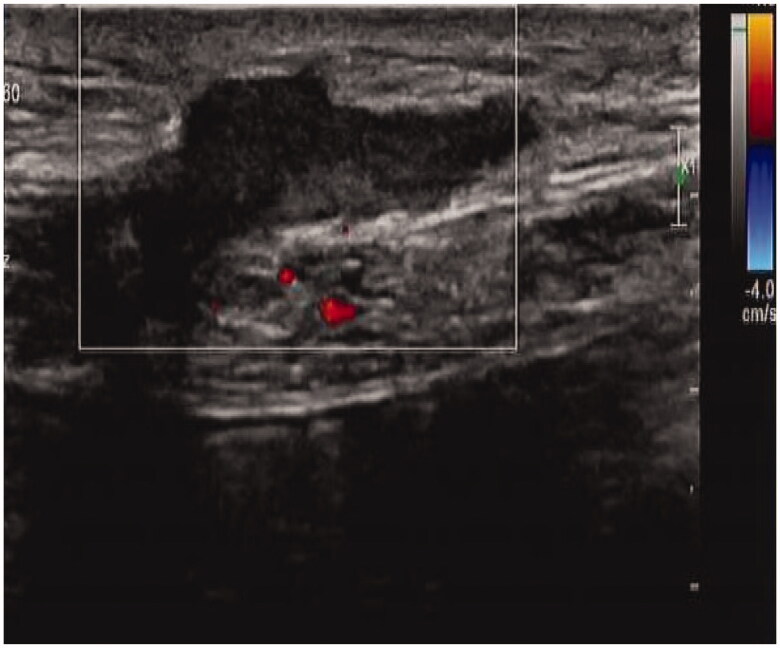
B-Mode Ultrasound Image of case 1.

A 36-year-old man was initiated on PD 2 years ago. He had a history of type I diabetes for >10 years and underwent kidney biopsy at our department 4 years ago. The pathological diagnosis was diabetic nephropathy. Owing to poor adequacy, he discontinued PD treatment and the PD catheter was removed by the ‘pull technique’ 1 year ago. After 4 months, secretions were found at the exit site of the original PD catheter, accompanied by pain and discomfort. Blood test results revealed the following: hemoglobin 121 g/L, blood cell count 8.32 × 10^9^/L, neutrophil percentage 73.4%, platelet count 153 × 10^9^/L, C-reactive protein 1.47 mg/L, and procalcitonin 0.37 ng/mL. Bacterial culture of the secretions revealed the presence of *Serratia marcescens*. B-mode ultrasound imaging revealed a heterogeneous mass in the left lower abdominal wall (Figure 1). We removed the mass by open surgery and administered piperacillin–sulbactam 2.5 g intravenously (IV) twice a day (BID) for 10 days. Subsequently, the patient was cured.

## Case 2

**Figure 2.**
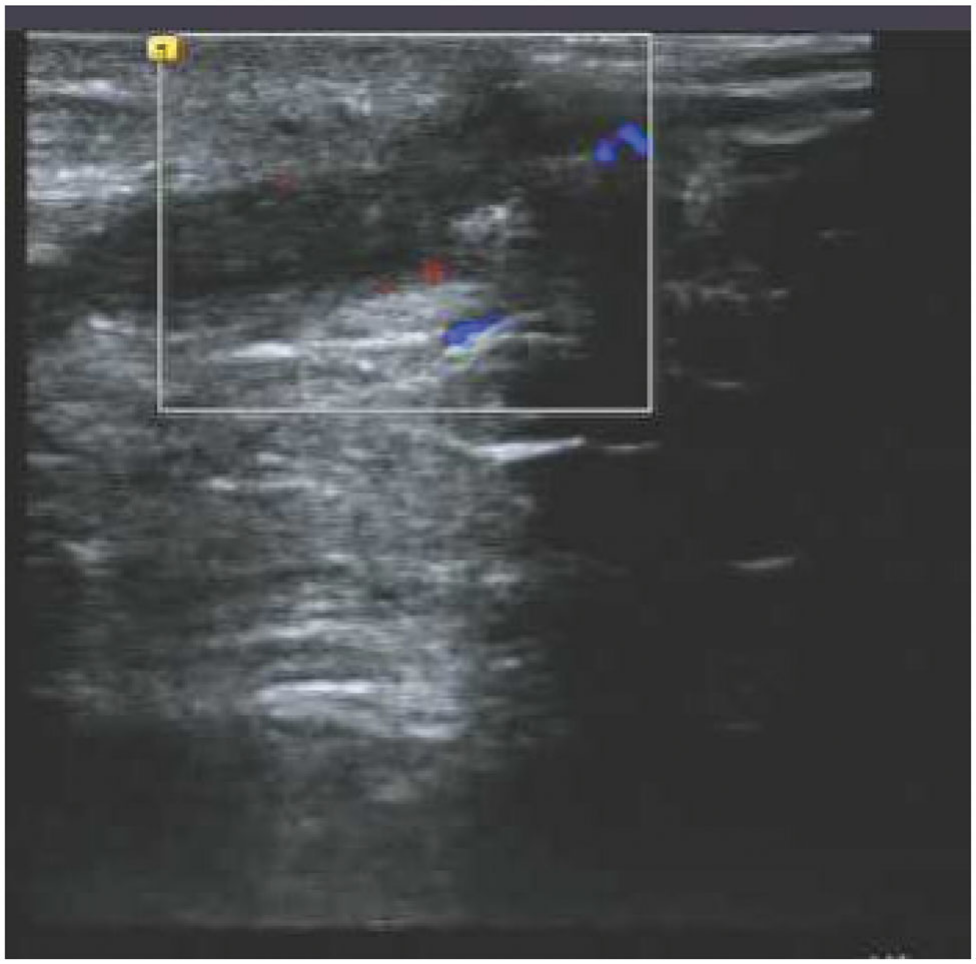
B-Mode Ultrasound Image of case 2 (a).

**Figure 3.**
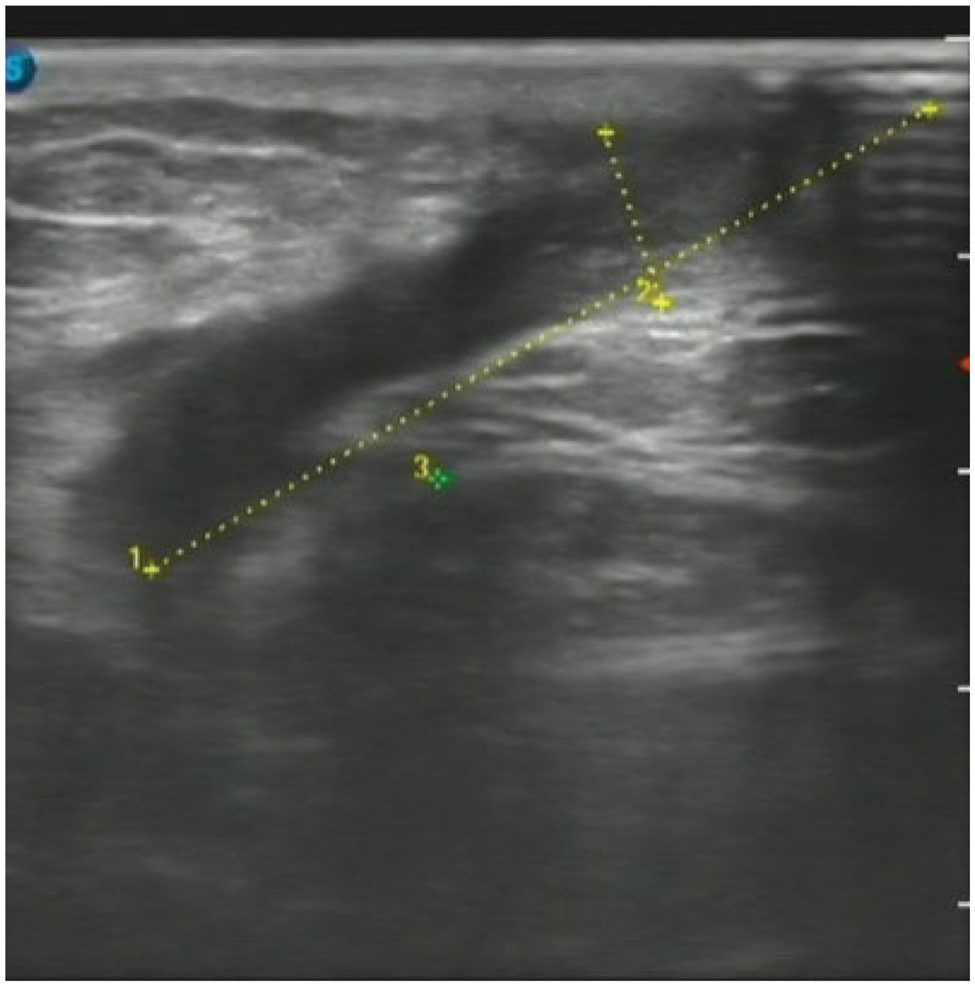
B-Mode Ultrasound Image of case 2 (b).

A 49-year-old man was initiated on PD treatment 6 years ago, and the primary causative disease was chronic glomerulonephritis. Owing to peritoneal ultrafiltration failure, PD treatment was stopped 6 months ago. PD catheter was removed by the ‘pull technique’. After 1 month, fluid exudation was noted at the exit site of the original PD catheter, accompanied by pain and no fever. Blood test results revealed the following: hemoglobin 118 g/L, white blood cell count 6.42 × 10^9^/L, neutrophil percentage 78.5%, platelet count 166 × 10^9^/L, C-reactive protein 2.56 mg/L, and procalcitonin 0.67 ng/mL. Bacterial culture of the secretion revealed the presence of *Pseudomonas putida*. B-mode ultrasound imaging revealed a heterogeneous echo mass in the left lower abdominal wall, measuring approximately 38 × 7 mm (Figure 2). We first administered piperacillin–sulbactam 2.5 g IV BID for 8 days without mass removal; however, the patient did not show any improvement, and B-mode ultrasound imaging indicated that the size of the mass in the left lower abdominal wall increased to approximately 45 × 8 mm (Figure 3). Abdominal wall mass resection was performed, and piperacillin–sulbactam was administered for 1 more week. Subsequently, the patient was cured.

## Case 3

**Figure 4.**
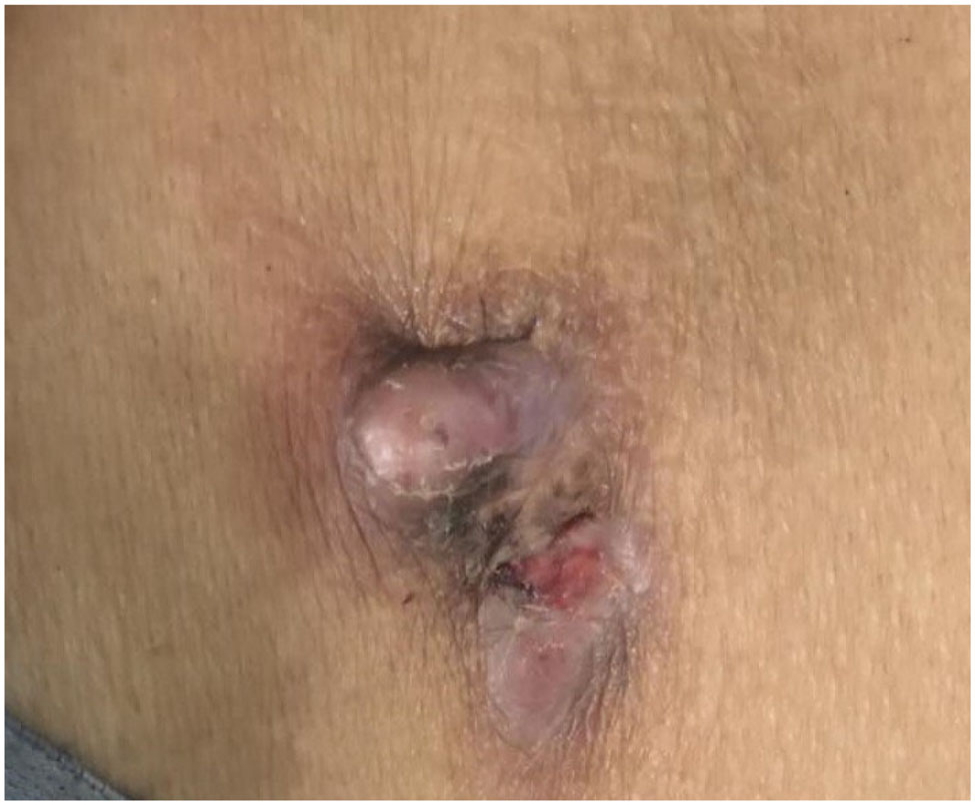
The infection of exit site of peritoneal dialysis catheter of case 3.

**Figure 5.**
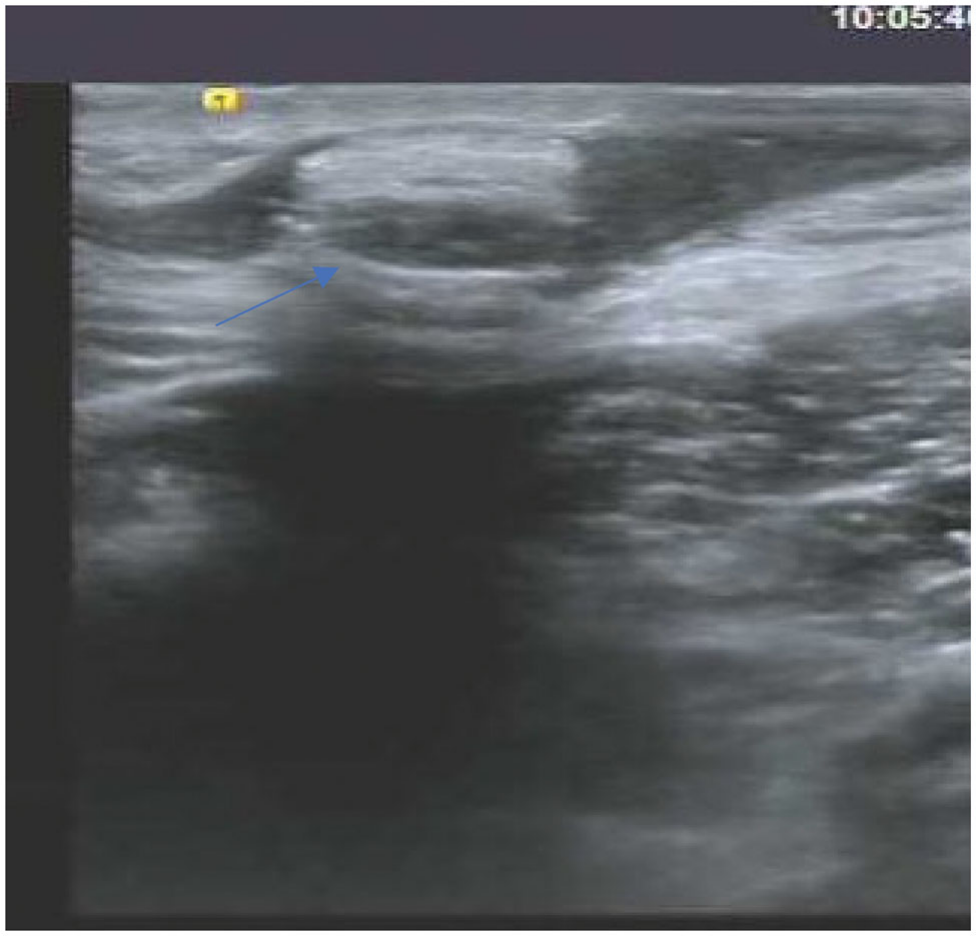
B-Mode Ultrasound Image of case 3.

**Figure 6.**
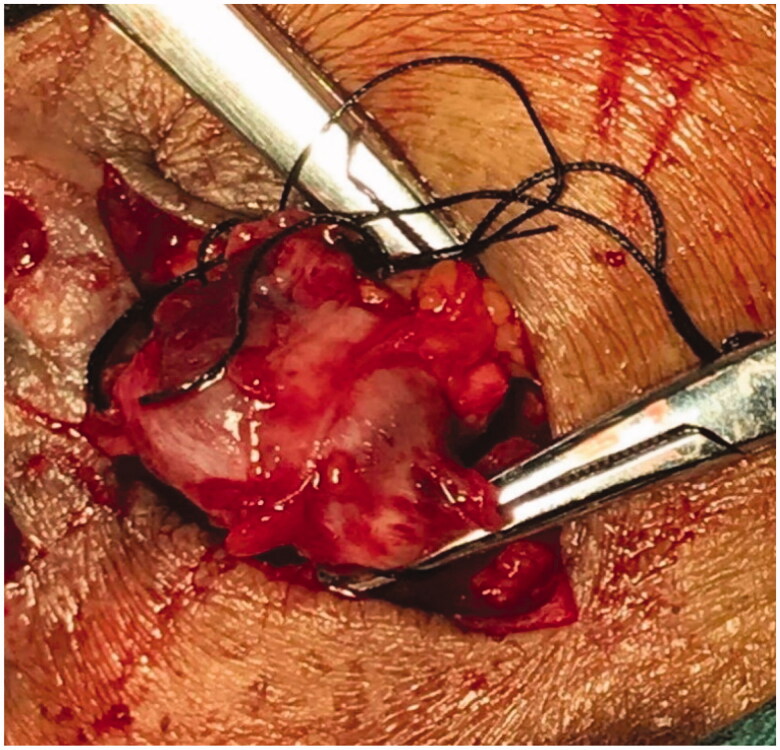
The mass removed by open surgery of case 3(a).

**Figure 7.**
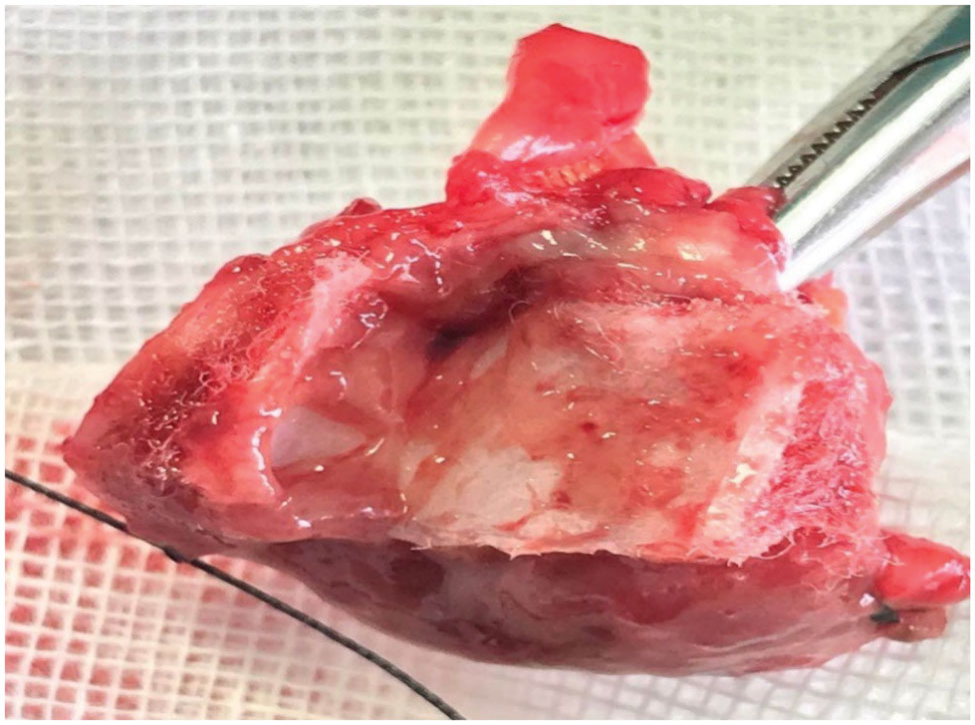
The mass removed by open surgery of case 3(b).

A 55-year-old man was initiated on PD treatment 5 years ago, and the primary causative disease was chronic glomerulonephritis. PD treatment was stopped 2 months ago because of fungal peritonitis. PD catheter was removed by the ‘pull technique’. After 3 weeks, the original PD catheter exit site was found to be red, swollen, and painful with purulent secretions (Figure 4). Blood test results revealed the following: hemoglobin 125 g/L, white blood cell count 5.43 × 10^9^/L, neutrophil percentage 58.7%, platelet count 216 × 10^9^/L, C-reactive protein 1.09 mg/L, and procalcitonin 1.09 ng/mL. Bacterial culture of the secretions revealed the presence of *Pseudomonas aeruginosa*. B-mode ultrasound imaging indicated a mixed mass in the left lower abdomen (Figure 5). We removed the mass by open surgery (Figures 6 and 7) and administered piperacillin–sulbactam 2.5 g IV BID for 10 days. Subsequently, the patient was cured.

PD is an effective renal replacement therapy for patients with end-stage kidney disease. When PD patients need to withdraw from PD treatment owing to reasons such as poor adequacy or refractory peritonitis, the PD catheter is usually removed. Previously, PD catheter was removed *via* open surgery; however, this method is more invasive, particularly when the deep cuff separation is prone to damage the rectus abdominis and abdominal artery, resulting in bleeding.

In recent years, several nephrologists are increasingly advocating the ‘pull technique’, described by Hakim et al. [1] for the first time in 1995. He successfully used the technique in 17 PD patients requiring catheter removal. Next, Quiroga et al. [2] used the ‘pull technique’ in 31 PD patients without peritonitis infection. Each patient was followed up for 2 years, and only one patient developed retained cuff-related infection. However, in the next 10 years, this technique was not used by nephrologists in China until Grieff et al. [3] reported the technique again in 2017. He used this technique in 46 patients of PD catheter removal, and only one patient exhibited retained cuff-related infection. He strongly recommended the use of the ‘pull technique’ for PD catheter removal. Subsequently, the technique was widely recognized by Chinese nephrologists.

Compared with open surgery, this technique is simple to perform, causes less surgical trauma, and requires less time. However, the PD catheter may rupture, and patients may experience acute pain when using the technique. The silicone PD catheters were hard enough, and there have been no cases of rupture at our center. Dezocine was administered intravenously and lidocaine was injected around the cuff to relieve the pain. A serious possible complication of this technique is retained cuff-related infection. The retained cuff is a foreign body in the abdominal wall, which may cause infection. Previous reports have indicated inconsistent incidence of retained cuff-related infection. For example, none of the 17 patients reported by Hakim et al. [1] exhibited infection, whereas only one patient exhibited retained cuff-related infection among the 31 patients reported by Quiroga et al. [2] and 46 patients reported by Grieff et al. [3]. However, Elkabir et al. [4] reported that the ‘pull technique’ was used in 62 PD patients, of whom 15 (24.2%) developed retained cuff-related infection. From the beginning of 2018 to the end of March 2020, a total of 30 PD catheters were removed by the ‘pull technique’ and 3 cases of retained cuff-related infections occurred, with an incidence rate of 10% at our center. Additionally, it is unclear whether there is an association between peritonitis and retained cuff-related infection. Some scholars may fear soft tissue infection caused by the outflow of infected fluid in patients with peritonitis. Except Case 3, the other two patients did not have peritonitis when the PD catheter was removed. Bacterial culture of secretions was different from the original peritonitis infection bacteria in Case 3. In addition, 8 PD patients who discontinued PD treatment owing to refractory peritonitis did not have retained cuff-related infection after PD catheter removal by the ‘pull technique’ at our PD center, suggesting that retained cuff-related infection is not related to the presence of peritonitis during PD catheter removal. Some scholars may be afraid that deep cuff was the source of the infection; however, we found that all the infections were related to superficial cuff. In Case 2, conservative anti-infective treatment alone was attempted for retained cuff-related infection, but it was ineffective. All three patients were cured after surgical resection together with anti-infective treatment. Therefore, we suggest that the basic principle is excision of the fistula canal, which can be the source of permanent infection and peritoneal content leakage. Moreover, all three patients had superficial cuff-related infections. Wang et al. [5] improved the ‘pull technique’ by isolating the superficial cuff and then using the ‘pull technique’ for PD catheter removal. He used the improved ‘pull technique’ in 24 patients and no cuff-related infection occurred. Superficial cuff separation is not difficult and it may be a better ‘pull technique’. In addition, the original exit site of PD catheter may be a site of bacterial invasion, which should be stitched.
